# A transgenic mouse model for tumour immunotherapy: induction of an anti-idiotype response to human MUC1

**DOI:** 10.1054/bjoc.2000.1431

**Published:** 2000-11

**Authors:** R W Wilkinson, E L Ross, A E Lee-MacAry, R Laylor, J Burchell, J Taylor-Papadimitriou, D Snary

**Affiliations:** Applied Development Laboratory, Imperial Cancer Research Technology, St. Bartholomew’s Hospital, London, EC1A 7BE; Breast Cancer Biology Group, Imperial Cancer Research Fund, Guy’s Hospital, London, SE1 9RT, UK

**Keywords:** immunotherapy, MUC1 transgenic mice, anti-idiotypic vaccines

## Abstract

MUC1 is a membrane bound, polymorphic epithelial mucin expressed at the luminal surface of glandular epithelium. It is highly expressed in an underglycosylated form on carcinomas and metastatic lesions and is, therefore, a potential target for immunotherapy of cancer. The monoclonal antibody HMFG1 binds the linear core protein sequence, PDTR, contained within the immunodominant domain of the tandem repeat of MUC1. The efficacy of murine and humanized HMFG1 (Ab1) used as an anti-idiotypic vaccine was examined in mice transgenic for human MUC1 (MUC1.Tg) challenged with murine epithelial tumour cells transfected with human MUC1. Humoral idiotypic cascade through Ab2 and Ab3 antibodies was observed in MUC1.Tg mice following multiple antibody inoculations in the presence of adjuvant. Impaired tumour growth at day 35 and highest Ab3 levels were found in mice that had received mHMFG1 with RAS adjuvant. However, comparison of Ab3 levels in individual mice with tumour size in all treatment groups did not show a correlation between smaller tumours and increased levels of anti-idiotype antibody. This suggests that the anti-tumour effects of anti-idiotype vaccination are not solely related to the induction of idiotypic antibody cascades and probably involve other mechanisms. © 2000 Cancer Research Campaign


British Journal of Cancer (2000) 83(9), 1202–1208
© 2000 Cancer Research Campaign
doi: 10.1054/ bjoc.2000.1431, available online at http://www.idealibrary.com on IDEAL®

### A transgenic mouse model for tumour immunotherapy: induction of an anti-idiotype response to human MUC1

**RW Wilkinson[1,2], EL Ross[1,2], AE Lee-MacAry[2], R Laylor[2], J Burchell[3], J Taylor-Papadimitriou[3] and D Snary[2]**

[2]Applied Development Laboratory, Imperial Cancer Research Technology, St. Bartholomew's Hospital, London EC1A 7BE; [3]Breast Cancer Biology Group, Imperial Cancer Research Fund, Guy's Hospital, London SE1 9RT, UK

**Summary** MUC1 is a membrane bound, polymorphic epithelial mucin expressed at the luminal surface of glandular epithelium. It is highly expressed in an underglycosylated form on carcinomas and metastatic lesions and is, therefore, a potential target for immunotherapy of cancer. The monoclonal antibody HMFG1 binds the linear core protein sequence, PDTR, contained within the immunodominant domain of the tandem repeat of MUC1. The efficacy of murine and humanized HMFG1 (Ab1) used as an anti-idiotypic vaccine was examined in mice transgenic for human MUC1 (MUC1.Tg) challenged with murine epithelial tumour cells transfected with human MUC1. Humoral idiotypic cascade through Ab2 and Ab3 antibodies was observed in MUC1.Tg mice following multiple antibody inoculations in the presence of adjuvant. Impaired tumour growth at day 35 and highest Ab3 levels were found in mice that had received mHMFG1 with RAS adjuvant. However, comparison of Ab3 levels in individual mice with tumour size in all treatment groups did not show a correlation between smaller tumours and increased levels of anti-idiotype antibody. This suggests that the anti-tumour effects of anti-idiotype vaccination are not solely related to the induction of idiotypic antibody cascades and probably involve other mechanisms. © 2000 Cancer Research Campaign

**Keywords:** immunotherapy; MUC1 transgenic mice; anti-idiotypic vaccines

In spite of being derived from self-tissue, tumours have been shown to express antigenic determinants, defined as tumour associated antigens (TAAs) which distinguish them from normal cells. TAAs are often expressed at high levels in tumours relative to normal tissue. Some TAAs are differentiation antigens that reflect the cell of origin, while others are molecules usually confined to normal undifferentiated cells during development and are termed onco-fetal antigens (Boon and Old, 1997). MUC1 falls into a third category of TAAs which are expressed at high levels on solid epithelial tumours and present an altered pattern of glycosylation (Burchell et al, 1987).

The MUC1 gene encodes the membrane bound, polymorphic epithelial mucin which contains an extended domain comprised of a variable number of a conserved 20 amino acid repeats (Gendler et al, 1987, 1988). Under normal circumstances, MUC1 is expressed on the luminal surface of most glandular epithelial cells but MUC1 has also been found on activated lymphocytes and has been proposed to be involved in the induction of T cell anergy (Agrawal et al, 1998b). The monoclonal antibody (mAb) HMFG1 binds to the linear core protein sequence, PDTR, contained within the immunodominant domain of the tandem repeats of MUC1 (Burchell and Taylor-Papadimitriou, 1993). HMFG1 has been used both to localize ovarian cancers by radioimmunoscintigraphy (Boyle et al, 1992) and for radiotherapy (Kosmas et al, 1998).

The unique complementarity determining regions (CDRs) within antibody variable regions can trigger a cascade of inter-reactive or anti-idiotypic antibodies. The idiotypic network comprise an antibody to antigen (Ab1) which in turn functions as an immunogen to elicit a second antibody (Ab2) specific to Ab1. In addition, a third wave of antibodies (Ab3) specific for Ab2 may be induced and these AB3 antibodies may cross-react with the original antigen (Jerne, 1974). Anti-idiotypic antibodies are thought to play a role in antibody regulation and provide extended antigenic exposure after clearance of the original foreign antigen (Bona, 1998). It has been hypothesized that prolonged anti-tumour immunity seen in some cancer patients and in animal models may reflect the development of idiotypic antibody networks (Mellstedt, 1995).

Although monoclonal antibody-based tumour immunotherapy has been studied for many years, animal models which allow human TAAs to be targeted in studies on immunotherapies involving anti-idiotype vaccination in tolerant, immunocompetent hosts have only recently been developed (Peat et al, 1992; Eades-Perner et al, 1994). The availability of mice transgenic for human MUC1, which are fully immunologically responsive and yet tolerant to human MUC1 (Peat et al, 1992; Rowse et al, 1998) now allows a detailed analysis of immune mechanisms which could be relevant to immunotherapy. Presented here is a pre-clinical study using murine and humanized HMFG1 as an idiotypic vaccine. The effects of route of antibody injection and various adjuvant formulations on idiotypic network formation and subsequent tumour clearance within a fully immunotolerant transgenic model are described.

### MATERIAL AND METHODS

### Animals

Human MUC1 transgenic mice (SacII) (CBA, H-$2^k$) were backcrossed with Balb/c (H-$2^d$) mice (non-Transgenic, non-Tg) to form a colony of SacII × Balb/c F1 (H-$2^k$, H-$2^d$) mice (MUC1.tg) (Peat

*Received 9 March 2000*
*Revised 23 June 2000*
*Accepted 28 June 2000*

*Correspondence to: RW Wilkinson*

[1] Authors contributed equally to the work.

et al, 1992; Graham et al, 1995). All MUC1.Tg and non-Tg mice used in experiments were 8–10-week-old females. Mice were housed and maintained in microisolator cages under pathogen-free conditions.

### Monoclonal antibodies

Antibodies used in this study were murine (m) (IgG1) and humanized (h) (IgG1) monoclonal anti-human MUC1 mAb, HMFG1 (Taylor-Papadimitriou et al, 1981; Burchell et al, 1983, 1987) and a murine (IgG1) and humanized (IgG1) monoclonal anti-carcinoembryonic antigen (CEA) antibody (clone PR1A3), as an isotype-matched control (i.c.) (Stewart et al, 1999). The hHMFG1 was obtained by transferring the complementarity determining regions (CDR) of the mouse antibody HMFG1 onto human framework regions (Verhoeyen et al, 1993). Antibodies were purified from cell culture supernatant by protein A affinity chromatography, gel filtration and ion exchange chromatography. Antibody used in immunization experiments was sterile and endotoxin free.

### Human MUC1$^+$ murine tumour cell line

The E4 cells used in these studies were derived from the murine mammary epithelial cell line, 4104.4 (Balb/c, H-2$^d$), which was transfected with the gene for human MUC1 (Lalani et al, 1991). Mice were injected subcutaneously (s.c.) with $2 \times 10^5$ cells in 100 μl of PBS to establish solid tumours. Tumours were measured every 3–4 days with vernier calipers by animal technicians who were unaware of the treatment and control groups. Tumour volumes were calculated as $(a \times b^2)/2$, where $a$ represents the largest and $b$ the smallest diameter. Human MUC1 expression was confirmed by flow cytometry on E4 cells before injection (FACScan, Becton Dickinson) using hHMFG1 followed by FITC-conjugated sheep anti-human IgG (Sigma). Expression of MUC1 in solid tumours was confirmed after 80 days of tumour growth by standard peroxidase anti-peroxidase immunohistochemical labelling of frozen tumour sections with hHMFG1.

### Immunizations and adjuvants

Animals received antibodies diluted in PBS with or without adjuvant in a final volume of 200 μl. Alum (Alhydrogel 2%, Superfos, Denmark) was mixed with antibody in a 1:1 ratio, RIBI Adjuvant System (RAS) contained synthetic trehalose dicorynomycolate (TDM) and monophoshoryl lipid A (MPL) in oil and Tween 80 and was prepared in accordance with the supplier's guidelines (R-700, RIBI Immunochem Research, Hamilton, USA). Provax (IDEC Pharmaceutical, San Diego, USA), which was a gift from Dr WJW Morrow, Queen Mary and Westfield College, London, had a composition of squalane (15% wt/vol), Tween 80 (0.6%) and pluronic acid L121 (3.75%) (Raychaudhuri et al, 1992). Antibody/adjuvant formulations were injected intraperitoneally (i.p.) or subcutaneously (s.c.) at two week intervals on four occasions over six weeks, and blood samples were obtained an hour before each injection. Four weeks after the final injection the animals were challenged with human MUC1$^+$ E4 cells.

### Detection of AB2 and AB3 antibodies by ELISA

Antibodies specific for HMFG1 were detected on 96-well microtitre plates coated with F(ab) fragments from HMFG1. F(ab) fragments were generated by digestion of HMFG1 (4 mg/ml in 0.1 M sodium acetate, pH 6.5, 3 mM EDTA, 50 mM DTT) with papain (0.2 mg/ml) for 4 hours at 37°C, and purified by Superose and MonoQ chromatography. A glutathione-S-transferase-MUC1 fusion protein (GST-MUC1) was employed for the detection of HMFG1 and Ab3 (Apostolopoulos et al, 1993). DNA coding for the seven tandem repeats of the 20 amino acid peptide epitope was inserted into the GST fusion vector pGEX-4T-1 (Pharmacia) and expressed in *E. coli* TG1 grown in L broth supplemented with 0.5 mM IPTG (Sigma). Following lysis with lysozyme in 1% Triton X-100 buffer, the fusion protein was affinity purified from supernatant using a glutathione-Sepharose 4B column (Pharmacia) and stored at 4°C in the presence of 2 mM glutathione until use.

Microtitre plates were coated overnight at 4°C with F(ab) fragments or GST-MUC1 (20 μg/ml in PBS) and blocked for 1 hour with 200 μl of 2% BSA in PBS. All plate washes and dilutions were performed with 0.02% Tween 20 in PBS. Serial dilutions of positive control antibodies, pre-treatment and post-treatment sera were prepared and 50 μl was added to each well. Antibody binding was detected with alkaline phosphatase conjugated anti-mouse pan-IgG (Sigma, UK) and p-nitrophenyl phosphate substrate (Sigma, UK). The subclasses of the Ab2 and Ab3 antibodies were determined using the above method with alkaline phosphatase conjugated anti-mouse IgG1, IgG2a IgG2b and IgG3 (Pharmingen, Cambridge Bioscience, UK) antibodies. Anti-MUC1 subclass control antibodies (Karsten et al, 1998) were kindly supplied by Dr Franz-Georg Hanisch (Institute of Biochemistry, Germany).

### Statistical methods

Group means were compared using Students' two-tailed, unpaired t-test for normal data sets and the Mann-Whitney U-test for non-parametric data. The degree of linear relationship between two data sets was determined by calculation of a Pearson correlation coefficient which has an absolute value between 0 and 1 (+ or –) with 1 indicating a perfect linear relationship. The Fisher's r to z transformation was performed to obtain a probability level ($P$ value) for the null hypothesis that the correlation is equal to zero. Probability values less than 0.01 were interpreted as significant. All statistics were performed using the SPSS statistical package.

### RESULTS

### Transgenic animals are tolerant to MUC1

Pre-existing antibody reactivity to MUC1 was not detected in either normal non-Tg mice ($n$ = 10) or MUC1.Tg mice ($n$ = 10). Following challenge with hMUC1-transfected E4 tumour cells, all non-Tg mice produced variable but significant levels of antibodies reactive with GST-MUC1, whereas no evidence of anti-MUC1 antibody production was observed in transgenic animals (Fig. 1A). Isotype analysis of the anti-MUC1 immunoglobulin response in the non-Tg mice demonstrated the presence of IgG1, IgG2a, IgG2b and IgG3 (Fig. 1B).

### Generation of anti-F(ab) and anti-MUC1 antibodies in MUC1.tg mice

Antibodies (Ab2) specific for the F(ab) fragment of mHMFG1 or hHMFG1 and GST-MUC1 (Ab3) were induced in MUC1.Tg mice

© 2000 Cancer Research Campaign

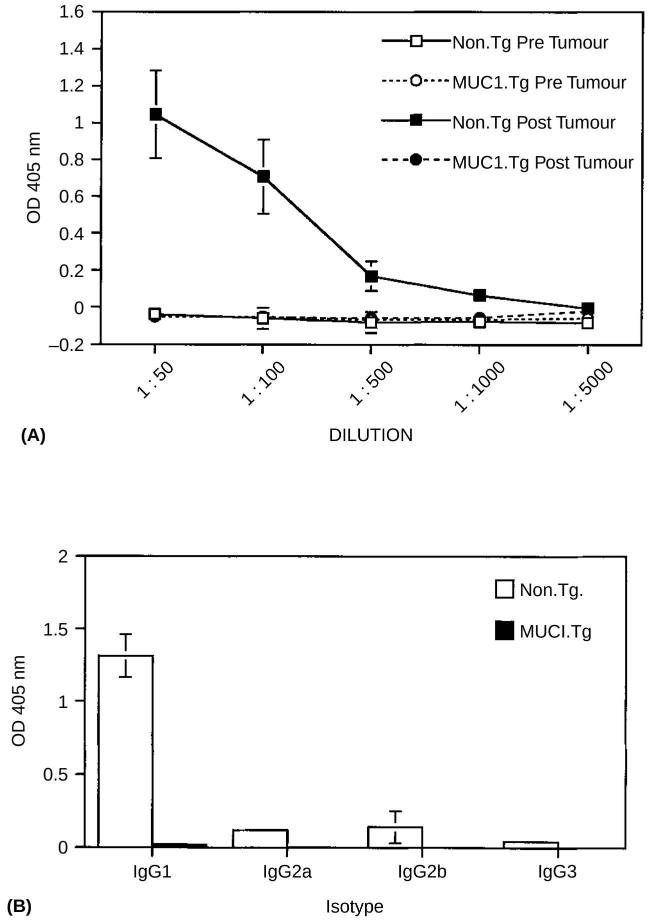

**Figure 1** ELISA analysis of anti-MUC1 antibody produced by non-Tg and MUC1-Tg mice after inoculation with MUC1 transfected tumour cells and pre-inoculation normal serum controls. (**A**) Total IgG levels, and (**B**) isotype analysis. Groups contained 10 mice and standard errors for mean absorbance values are shown

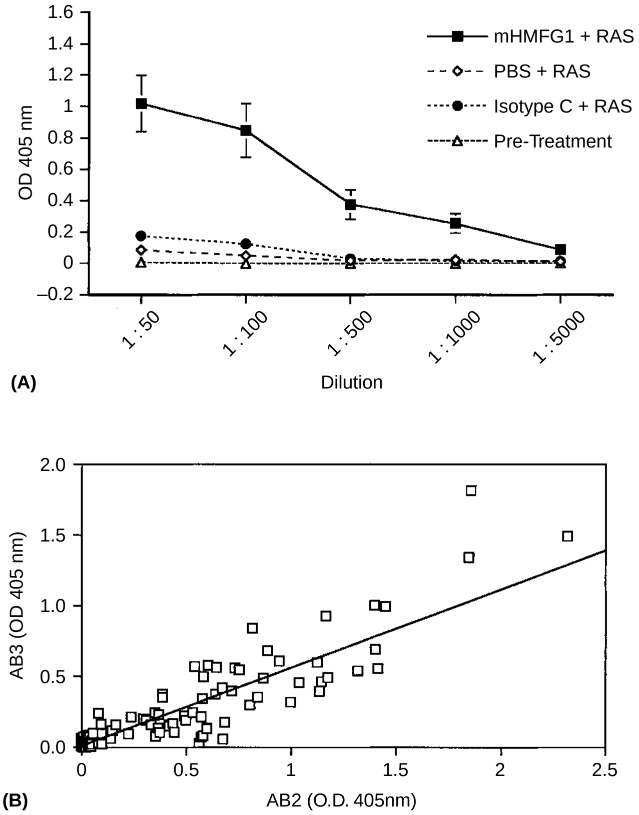

**Figure 2** MUC1.Tg mice were injected i.p. on three occasions, two weeks apart, with mHMFG1 + RAS. (**A**) Titration of anti-MUC1 antibody, control sera include pre-immune sera groups immunized with either an isotype-matched control antibody PR1A3 (anti-CEA antibody) or PBS. Groups contained 8 mice and standard errors for mean absorbance values are shown.
(**B**) Positive correlation between Ab2 and Ab3 levels in immunized MUC1.Tg mice. MUC1.Tg mice (10/group) were inoculated i.p. with mHMFG1 with and without RAS adjuvant, or with murine isotype control antibody with and without RAS, or PBS with and without RAS adjuvant, on four occasions two weeks apart. Sera samples were assayed at dilutions of 1/50

following serial injections of murine or humanized HMFG1 with or without adjuvant. Highest levels of anti-MUC1 Ab3 antibodies were detected after immunization with mHMFG1 with RAS adjuvant which contrasted with the low to negligible Ab3 levels produced following injection with isotype and diluent controls (Fig. 2). Sera from immunized mice did not bind to a F(ab) fragment of an isotype-matched control antibody or a control antigen (CEA) (data not shown). This confirms that the responses detected were true Ab2 and Ab3 responses specific for HMFG1 and MUC1 respectively. The magnitude of anti-idiotypic and anti-anti-idiotypic responses was proportional to the amount of HMFG1 injected. Mice which received 100 μg of HMFG1 produced higher levels of Ab2 and Ab3 antibodies than those which received 10 μg or 40 μg of HMFG1 (data not shown). Serum Ab2 levels always exceeded Ab3 levels and a positive correlation was observed between Ab2 and Ab3 (Pearson correlation coefficient: non-Tg $r^2$ = 0.502; MUC1.Tg $r^2$ = 0.806; $P < 0.001$) (Fig. 2B).

### Factors effecting the generation of Ab2 and Ab3 antibodies

The levels of Ab2 and Ab3 antibodies obtained were influenced by the number of injections, the adjuvant used and the route of administration. HMFG1 was administered on four occasions at least two weeks apart, either i.p. or s.c., in combination with three different adjuvants: Alum, Provax and RAS (Fig. 3A and B). Highest levels of Ab2 and Ab3 antibody were obtained following three injections of mHMFG1 with RAS adjuvant (Fig. 3C). Any contribution of residual circulating HMFG1 to the Ab3 levels was thought to be minimal since the half-life of the antibody was 7 days (data not shown). However, residual antibody could account for the slightly lower values measured four weeks after the last injection in comparison to values seen two weeks after the third injection.

A comparison of adjuvant and route of immunization demonstrated that i.p gave better Ab3 responses than s.c. and these responses were heightened by the addition of an adjuvant (Fig. 3). The potentiation of Ab3 responses with adjuvant on s.c inoculation was much less evident. The optimal conditions for Ab3 induction were i.p immunization with RAS in preference to alum or Provax. This was achieved with antibody concentrations of 100 μg per

© 2000 Cancer Research Campaign

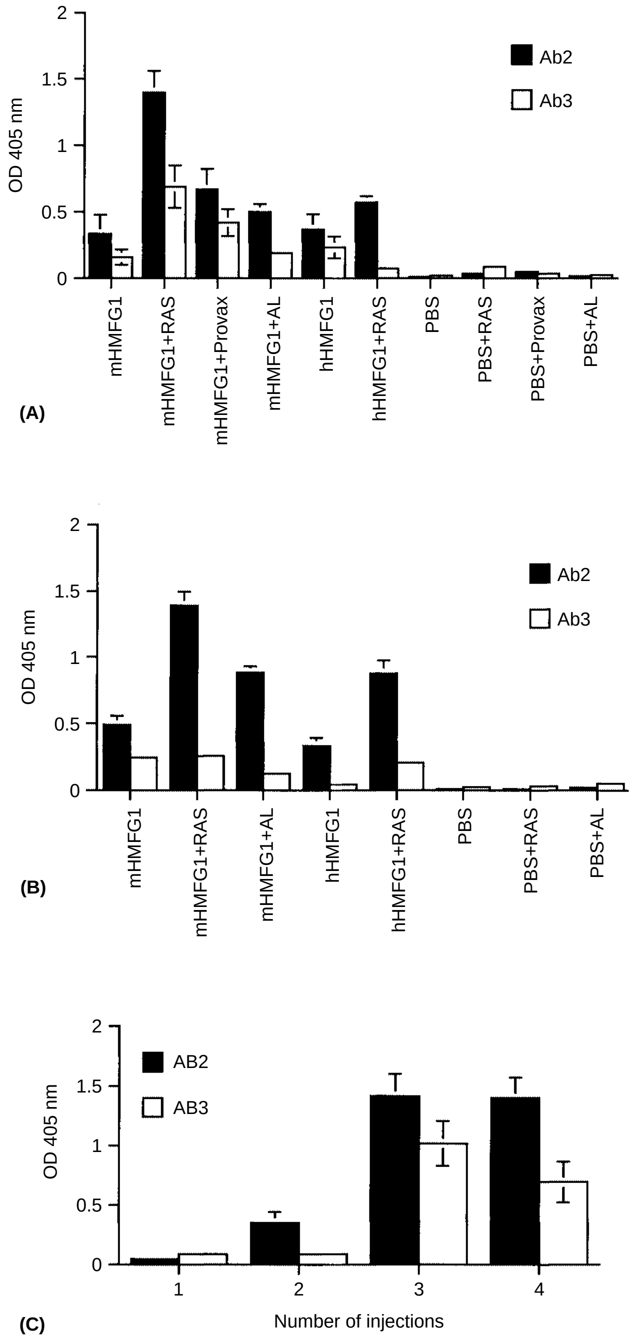

**Figure 3** The effect of adjuvant and number of inoculations on the production of Ab2 and Ab3 antibodies by MUC1.Tg mice. Mice (10/group) received four injections either i.p. (**A**), or s.c. (**B**), of 100 μg murine (m) or human (h) HMFG1 with or without adjuvant (RAS, Provax, Alum). (**C**) Ab2 and Ab3 levels increase with the number of HMFG1 injections. MUC1.Tg mice (10/group) were injected i.p. with 100 μg of mHMFG1 + RAS. Inoculations were two weeks apart and sera samples were taken two weeks after injection, except for the final samples which were taken four weeks after the final injection. Sera samples were assayed at 1/50 dilution and mean absorbance values ± standard errors are shown.

dose, and lesser responses occurred with lower doses of antibody (data not shown). Ab2 levels were much less influenced by route and equivalent values were found for both s.c and i.p. immunization. Significantly, a comparison of murine and humanized

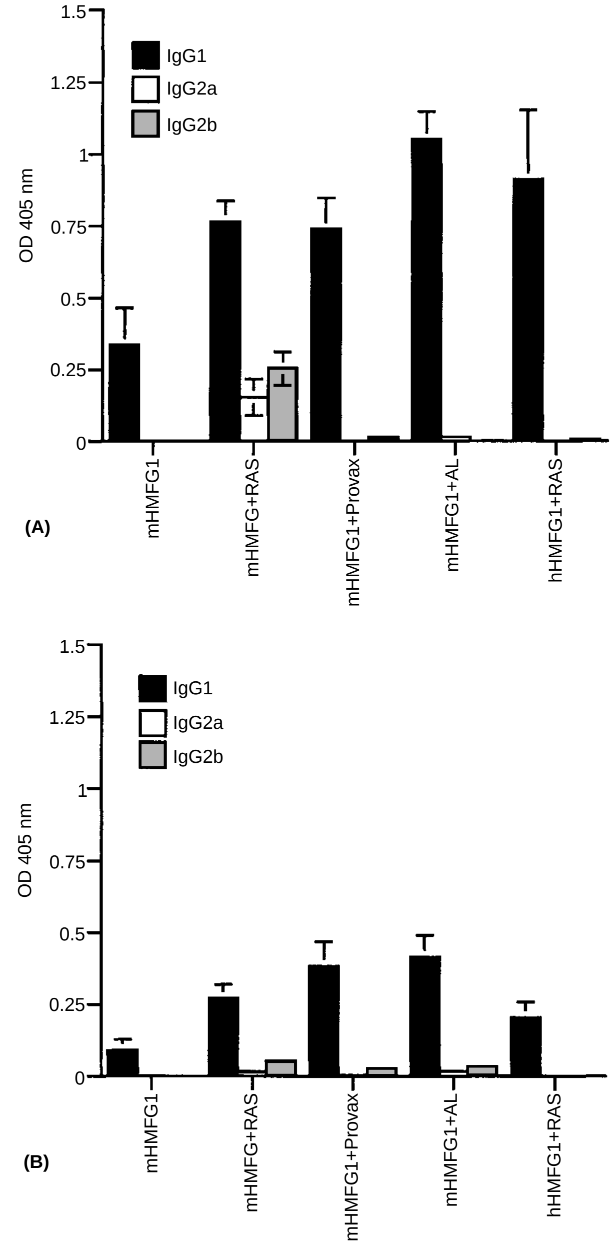

**Figure 4** The isotype of the Ab2 (**A**) and Ab3 (**B**) antibodies generated in MUC1.Tg (10/group) mice after four i.p. immunizations with either murine or human HMFG1 with or without adjuvant (RAS; Provax; Alum). Mean absorbance values ± standard errors are shown

HMFG1 given with RAS demonstrated that the murine antibody in a mouse was a more effective inducer of an Ab3 response than the humanized antibody (Fig. 3).

### Effect of adjuvant on anti-MUC1 antibody (Ab3) isotype

The isotype profile was determined for the Ab2 and Ab3 antibodies produced in MUC1.Tg mice after i.p. immunization on four occasions. The isotype of the Ab2 induced was IgG1 in the majority of cases (Fig. 4). However, adjuvant influenced the isotype profile in that i.p. injection of mHMFG1 with RAS produced IgG2a and IgG2b Ab2 antibodies. The Ab3 isotypes from mice which received mHMFG1 plus adjuvant were almost

© 2000 Cancer Research Campaign

**Table 1** AB3 levels and tumour size in MUC1.Tg mice following mHMFG1 treatment

| Treatment | AB3 ($OD_{405nm}$)[a] | Tumour size ($cm^3$)[b] |
|---|---|---|
| mHMFG1 | 0.156 ± 0.057 | 0.132 ± 0.041 |
| mHMFG1 + RAS | 0.695 ± 0.160 | 0.100 ± 0.029 |
| ic.[c] mIgG1 | 0.088 ± 0.027 | 0.199 ± 0.031 |
| ic. mIgG1 + RAS | 0.169 ± 0.022 | 0.311 ± 0.035 |
| PBS | 0.021 ± 0.005 | 0.211 ± 0.032 |
| PBS + RAS | 0.088 ± 0.027 | 0.278 ± 0.033 |

[a] Mean (*n* = 10) ± s.e. AB3 levels in 1:50 diluted sera obtained 4 weeks after the final antibody injection and before challenge with E4 cells. [b] Mean (*n* = 10) ± s.e. tumour volume was calculated 35 days after challenge with $2 \times 10^5$ E4 cells. [c] i.c.: isotype-matched control.

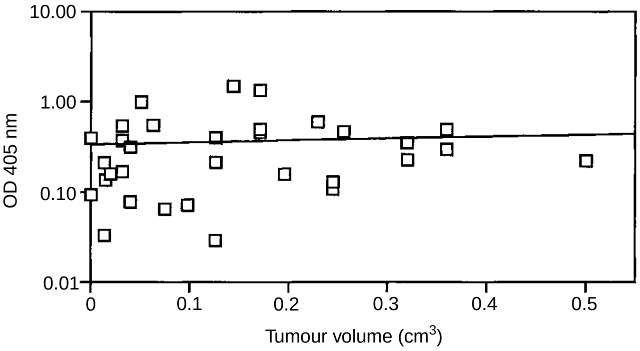

**Figure 5** Comparison of tumour size and AB3 levels. MUC1.Tg mice received four i.p. injections of murine HMFG1 with or without adjuvant (RAS; Provax; Alum) followed by one s.c. injection of $2 \times 10^5$ tumour cells. Levels of serum anti-MUC1 Ab3 antibodies were assessed immediately before administration of E4 cells. Tumour volumes 35 days following cell injection are compared with Ab3 antibody levels for individual mice exclusively IgG1 with only very low levels of IgG2a and G2b detected which did not vary significantly for the different adjuvants used. Mice receiving hHMFG1 plus RAS only produced IgG1 Ab2 and Ab3 antibodies. No IgG3 Ab2 or Ab3 antibodies were detected in any of the samples.

### Tumour development following immunization schedule

Tumour growth kinetics and survival were determined for immunized mice by giving a single s.c. inoculation of $2 \times 10^5$ E4 tumour cells four weeks after the final immunization. All mice developed solid, highly vascularized tumours within 28 days. Tumour size at 35 days was compared between mice which had received i.p. injections of mHMFG1 with or without RAS, murine isotype control antibody with or without RAS and PBS with or without RAS. Although the mean tumour size in mice immunized with mHMFG1 was smaller than those from mice immunized with diluent (PBS) and isotype control antibody, the difference was not significant (mHMFG1/PBS, $P$ = 0.198; mHMFG1/i.c., $P$ = 0.1849). In contrast, tumours grown in mice which received mHMFG1 + RAS were significantly smaller than all control groups (mHMFG1 + RAS/PBS, $P$ = 0.015, mHMFG1 + RAS/PES + RAS, $P$ = 0.003, mHMFG1 + RAS/i.c., $P$ = 0.017, mHMFG1 + RAS/i.c. + RAS, $P$ = 0.001).

Ab3 levels in the mHMFG1 + RAS treatment group was significantly higher than the control groups (mHMFG1 + RAS/PBS, $P$ = 0.001, mHMFG1 + RAS/PBS + RAS, $P$ = 0.002, mHMFG1 + RAS/i.c., $P$ = 0.002, mHMFG1 + RAS/i.c. + RAS, $P$ = 0.006). Ab3 levels generated in the mHMFG1 treatment group were significantly higher when compared with the diluent control (mHMFG1/PBS, $P$ = 0.03) but no statistically significant differences were found between mHMFG1 and the other control groups (mHMFG1/PBS + RAS, $P$ = 0.275, mHMFG1/i.c., $P$ = 0.301, mHMFG1/i.c. + RAS, $P$ = 0.840) (Table 1). In spite of the fact that smallest tumours and highest AB3 levels occurred in the HMFG1 + RAS group, when individual samples from this treatment group were examined, a negative correlation did not exist between tumour size and AB3 levels. Furthermore, when tumour size at day 35 and AB3 levels from individual mice from all treatment groups (mHMFG1 alone and with adjuvants) were compared, no inverse correlation was found (Pearson correlation coefficient $r^2$ = 0.084; $P$ = 0.685) (Fig. 5). When the effect of adjuvant within the controls was analysed no statistically significant difference was observed in tumour growth (PBS/PBS + RAS, $P$ = 0.130, i.c./i.c. + RAS, $P$ = 0.053). Comparison of Ab3 levels between control groups with and without RAS revealed that the groups given the adjuvant had significantly higher Ab3 levels than non-RAS growth (PBS/PBS + RAS, $P$ = 0.0003, i.c./i.c. + RAS, $P$ = 0.0156).

### DISCUSSION

A number of groups have explored the possibility of utilizing anti-idiotypic vaccines for the treatment of cancer either as antibodies specific for TAA (Ab1) or 'internal image' antibodies which mimic TAAs (Ab2) (Mellstedt, 1995; Bona, 1998). Some success has been attributed to anti-Id therapy, for instance, patients with Dukes' C colorectal cancer receiving adjuvant treatment with the murine mAb 17-1A (anti-Ep-CAM) showed a 30% improvement in overall survival and an equivalent reduction in distant metastatic recurrence (Riethmuller et al, 1994). This study and others have established the safety of an anti-id approach, but the immune and anti-tumour responses stimulated by these therapies have yet to be clearly defined. This in part is because studying anti-idiotype vaccination against human TAAs has been difficult in animal models since a fully competent immune system is needed.

MUC1 transgenic mice appear to be fully tolerant to human MUC1 in that they do not develop a humoral response to the antigen despite it being aberrantly glycosylated within the tumour microenvironment (Graham et al, 1996). This result was interesting since some humans have been reported to produce antibodies to under-glycosylated MUC1 (von Mensdorff-Pouilly et al, 1996). To more accurately mimic tumour growth in humans, an adenocarcinoma line was chosen (Miller et al, 1983; Lalani et al, 1991). This line not only grows slowly in vivo (tumour related death does not take place until 40 to 80 days after inoculation of $2 \times 10^5$ cells) but expresses low levels of class I antigen at its surface. This makes the tumour line transfected with MUC1 analogous to a poorly differentiated, invasive breast carcinoma (Garrido et al, 1993). A key factor in the use of this tumour is that even after 80 days growth in vivo tumour cells maintain surface expression of human MUC1 and thus remain good targets for immunotherapy.

Vaccination with HMFG1 can break tolerance to MUC1 in the MUC1.Tg mice with the appearance of Ab2 and Ab3 antibodies. These responses were influenced by the number of injections, the route of injection, and by the adjuvant used. Using this model, we found that induction of significant anti-MUC1 Ab3 antibody

 © 2000 Cancer Research Campaign responses required at least three 100 μg doses of antibody, that i.p. immunization was better than s.c., and that a strong adjuvant gave a heightened response. Interestingly, higher levels of AB3 were obtained using murine as compared to humanized antibody. This suggests that homologous rather than heterologous antibody would be the better antibody to use in an idiotypic vaccination programme. The superior performance of murine antibody in mouse may be influenced by the strong MAHA (mouse anti-human antibody) response given when the heterologous antibody is used (data not shown), which may compete with or inhibit the idiotypic cascade.

TAAs targeted for anti-idiotype immunotherapy in clinical trials include melanoma-associated chondroitin sulphate proteoglycan (MPG) (Pride et al, 1998), carcinoembryonic antigen (CEA) (Foon et al, 1997) and the disialogangliosides GD2 (Chapman et al, 1994) and GD3 (Foon et al, 1998). The adjuvants used in these studies have been shown to be effective in increasing humoral responses. For example, the adjuvant QS-21 administered with an anti-GD3 Ab2 antibody induced Ab3 antibodies in all patients studied. Within this group of 12 melanoma patients, one made a complete clinical response with soft tissue resolution, while six had stable disease ranging from 9 to 23 months (Foon et al, 1998). The most effective adjuvant in our study, RAS, is a mycobacterium derivative in an oil and water mix which has previously been used in human cancer vaccine trials and has been reported to augment protective antibody responses (Daly and Long, 1996; Miles et al, 1996). It has previously been shown that type 1 responses are characterized by the production of IgG2a antibodies, whereas IgG1 is present in both type 1 and type 2 immune responses (Rodolfo et al, 1998). Immunization with HMFG1 in transgenic mice resulted in Ab2 and Ab3 antibodies which were predominantly IgG1 and the presence of RAS induced small quantities of IgG2a and IgG2b Ab2 antibodies.

When the antibody responses for individual animals were compared, it was clear that there was a direct correlation between the level of Ab2 and Ab3 in that a strong Ab2 response was associated with a strong Ab3 response. Highest Ab3 levels and slowest tumour growth occurred in MUC1.tg mice after vaccination with murine HMFG1 plus RAS. However, this inhibition could not be attributed solely to the induction of anti-MUC1 Ab3 antibodies in an idiotypic cascade since no significant correlation between Ab3 levels and tumour retardation was observed when the mice were examined on an individual level. In addition to humoral responses, it has been suggested that anti-Id therapy can activate cellular immunity to TAAs (Pride et al, 1998; Ruiz et al, 1998). This may explain why some mice within the murine HMFG1 plus RAS group, despite having low Ab3 antibody levels, were better protected against tumour challenge than others with high Ab3 levels.

Regardless of the type of treatment administered in this study, an induced anti-MUC1 response resulting from idiotypic vaccination did not confer complete protection when mice were challenged with the syngeneic $hMUC1^+$ E4 tumour line. The contribution of anti-MUC1 humoral response in MUC1 tumour biology is controversial. Mice with high Ab3 levels did not appear to have a significant survival advantage, and passive transfer of polyclonal sera from a non.Tg mouse primed with $hMUC1^+$ B16 cells into MUC1.Tg mice did not confer protection against transplanted $hMUC1^+$ tumours (Tempero et al, 1999). In humans, circulating anti-MUC1 IgG antibodies have been associated with a good prognosis in early stage breast cancer patients, but not with advanced stage disease at first diagnosis (von Mensdorff-Pouilly et al, 2000). The disparity between these two results may reflect tumour load.

As the immune response to solid tumours is dissected, it has become evident that tumours employ a myriad of escape mechanisms which thwart an active immune response. Underglycosylation of MUC1 and its elevated expression in epithelial malignancies make tumour-associated MUC1 a reasonable target for cancer immunotherapy (Agrawal et al, 1998a), but it is evident that production of an anti-idiotype response alone is insufficient to mount a therapeutic anti-tumour effect. This observation is further complicated by the recent reports that MUC1 is expressed and secreted by activated T cells and may have immunoregulatory properties (Agrawal et al, 1998b). MUC1 has also been shown to induce T cell anergy and interfere with NK-mediated lysis (Zhang et al, 1997) which could have a significant impact on tumour therapy and may even help to protect the $MUC1^+$ tumour from cell-mediated attack.

The most effective immunotherapies are likely to employ more than one aspect of the immune system. Activated $CD4^+$ T cells (Hung et al, 1998) and APC/tumour cell fusions have recently been shown to be important in primary stimulation of an anti-tumour response (Gong et al, 1998). Furthermore, CTLs effectively protected animals from tumour challenge, but both antibodies and CTLs were required to eliminate established tumours (Vasovic et al, 1997). It is possible that anti-MUC1 antibodies produced by an idiotyic cascade could provide protection against tumour growth in minimal residual disease, but this may rely upon synergy with effector cells.

### ACKNOWLEDGEMENTS

Financial support by Antisoma Plc is gratefully acknowledged. We thank Gary Martin and Barbara Turner, ICRF Biological Resources Unit, for their skilled technical support, Dr Gillian Lewis, Biotherapeutics Development Unit, for preparation of the antibodies, Heather Band for ELISA development, and Dr Rosalind Graham, ICRF Breast Cancer Biology Group, for advice on the MUC1 tumour model. Statistical analyses were performed by Marialena Trivella from the ICRF Medical Statistics Group. We are grateful to Dr Franz-Georg Hanisch for supplying anti-MUC1 subclass control antibodies.

### REFERENCES

Agrawal B, Gendler SJ and Longenecker BM (1998a) The biological role of mucins in cellular interactions and immune regulation: prospects for cancer immunotherapy. *Molecular Medicine Today* 397–403

Agrawal B, Krantz MJ, Reddish MA and Longenecker BM (1998b) Cancer-associated MUC1 mucin inhibits human T-cell proliferation, which is reversible by IL-2. *Nature Medicine* **4**: 43–49

Apostolopoulos V, Xing PX, Trapani JA and McKenzie IF (1993) Production of anti-breast cancer monoclonal antibodies using a glutathione-S-transferase-MUC1 bacterial fusion protein. *Br J Cancer* **67**: 713–720

Bona CA (1998) Idiotype vaccines: forgotten but not gone. *Nature Medicine* **4**: 668–669

Boon T and Old LJ (1997) Tumor antigens. *Current Opinion in Immunology* **9**: 681–683

Boyle CC, AJ, P and Mather S (1992) The mechanism of hepatic uptake of a radiolabelled monoclonal antibody. *Int J Cancer* **50**: 912–917

Burchell J and Taylor-Papadimitriou J (1993) Effect of modification of carbohydrate side chains on the reactivity of antibodies with core-protein epitopes of the MUC1 gene product. *Epithelial Cell Biol* **2(4)**: 155–162

© 2000 Cancer Research Campaign 

Burchell J, Durbin H and Taylor-Papadimitriou J (1983) Complexity of expression of antigenic determinants recognised by monoclonal antibodies HMFG-1 and HMFG-2 in normal and malignant human epithelial cells. *J Immunol* **131**: 508–513

Burchell J, Gendler SJ, Taylor-Papadimitriou J, Girling A, Lewis A, Millis R and Lamport D (1987) Development and characteristics of breast cancer reactive monoclonal; antibodies directed to the core protein of the human milk mucin. *Cancer Res* **47**: 5476–5482

Chapman PB, Livingston PO, Morrison ME, Williams L and Haughton AN (1994) Immunization of melanoma patients with anti-idiotypic monoclonal antibody BEC2 which mimics GD3 ganglioside: pilot trials using no immunological adjuvant. *Vaccine Res* **3**: 59–69

Daly TM and Long CA (1996) Influence of adjuvants on protection induced by a recombinant fusion protein against malarial infection. *Infect Immun* **64**: 2602–2608

Eades-Perner E-P, van der Putten H, Hirth A, Thompson J, Neumaier M, von Kleist S and Zimmermann W (1994) Mice transgenic for human carcinoembryonic antigen gene maintain its spatiotemporal expression pattern. *Cancer Res* **54**: 4169–4176

Foon KA, John WJ, Chakraborty M, Sherratt A, Garrison J, Flett M and Bhattacharya-Chatterjee M (1997) Clinical and immune responses in advanced colorectal cancer patients treated with anti-idiotype monoclonal antibody vaccine that mimics the carcinoembryonic antigen. *Clin Cancer Res* **3**: 1267–1276

Foon KA, Sen G, Hutchins L, Kashala OL, Baral R, Banerjee M, Chakraborty M, Garrison J, Reisfeld RA and Bhattacharaya-Chatterjee M (1998) Antibody responses in melanoma patients immunized with an anti-idiotype antibody mimicking disialoganglioside GD21. *Clin Cancer Res* **4**: 1117–1124

Garrido F, Cabrera T, Concha A, Glew S, Ruiz-Cabello F and Stern PL (1993) Natural history of HLA expression during tumour development. *Immunol. Today* **14**: 491–499

Gendler SJ, Burchell J, Duhig T, Lamport D, White R, Parker M and Taylor-Papadimitriou J (1987) Cloning of partial cDNA encoding differentiation and tumour-associated mucin glycoprotein expressed by human mammary epithelium. *Proc Natl Acad Sci USA* **84**: 6060–6064

Gendler SJ, Taylor-Papadimitriou J, Duhig T, Rothbard J and Burchell J (1998) A highly immunogenic region of human polymorphic epithelial mucin expressed by carcinomas is made up of tandem repeats. *J Biol Chem* **263**: 12820–12823

Graham RA, Stewart LS, Peat NP, Beverley P and Taylor-Papadimitriou J (1995) MUC1-based immunogens for tumour therapy: development of murine model systems. *Tumour Targeting* **1**: 211–221

Graham RA, Burchell JM and Taylor Papadimitriou J (1996) The polymorphic epithelial mucin: potential as an immunogen for a cancer vaccine. *Cancer Immunol Immunother* **42**: 71–80

Jerne NK (1974) Towards a network theory of the immune system. *Ann Immunol (Paris)* **125C**: 373–389

Karsten U, Diotel C, Klich G, Paulsen H, Goletz S, Muller S and Hanisch F-H (1998) Enhanced binding of antibodies to the DTR motif of MUC1 tandem repeat peptide is mediated by site-specific glycosylation. *Cancer Res* **58**: 2541–2549

Kosmas C, Kalofonos HP, Hird V and Epenetos AA (1998) Monoclonal antibody targeting of ovarian carcinoma. *Oncology* **55**: 435–446

Lalani EN, Berdichevsky F, Boshell M, Shearer M, Wilson D, Gendler SJ and Taylor-Papadimitriou J (1991) Expression of the gene encoding for human mucin in mouse mammary tumour cells can effect tumorigenicity. *J Biol Chem* **266**: 15420–15426

Mellstedt FH (1995) Anti-idiotypes and cancer. *The Cancer Journal* **8**: 181–184

Miles DW, Towlson KE, Graham R, Reddish M, Longenecker BM, Taylor-Papadimitriou J and Rubens RD (1996) A randomised phase II study of sialyl-Tn and DETOX-B adjuvant with or without cyclophosphamide pretreatment for the active specific immunotherapy of breast cancer. *Br J Cancer* **74**: 1292–1296

Miller FR, Miller BE and Heppner GH (1983) Characterization of metastatic heterogeneity among subpopulations of a single mouse mammary tumor: heterogeneity in phenotypic stability. *Invasion Metastasis* **3**: 22–31

Peat N, Gendler SJ, Lalani EN, Duhig T and Taylor-Papadimitriou J (1992) Tissue specific expression of a human polymorphic epithelial mucin (MUC1) in transgenic mice. *Cancer Res* **52**: 1954–1960

Pride MW, Shuey S, Grillo-Lopez A, Braslawsky G, Ross M, Legha SS, Eton O, Buzaid A, Ioannides C and Murray JL (1998) Enhancement of cell-mediated immunity in melanoma patients immunized with murine anti-idiotypic monoclonal antibodies (MELIMMUNE) that mimic the high molecular weight proteoglycan antigen. *Clin Cancer Res* **4**: 2363–2370

Raychaudhuri S, Tonks M, Carbone F, Ryskamp T, Morrow W.J.W. and Hanna N (1992) Induction of antigen-specific class 1-restricted cytotoxic T cells by soluble proteins in vivo. *Proc Natl Acad Sci USA* **89**: 8308–8312

Riethmuller G, Schneider-Gadicke E, Schlimok G, Schmiegel W, Raab R, Hofken K, Gruber R, Pichlmaier H, Hirche H, Pichlmayr R, Buggisch P, Witte J and Group TGCA-AS (1994) Randomised trial of monoclonal antibody for adjuvant therapy of resected Dukes' C colorectal carcinoma. *Lancet* **343**: 1177–1183

Rodolfo M, Melani C, Zilocchi C, Cappetti B, Luison E, Arioli I, Parenza M, Canevari S and Colombo MP (1998) IgG2a induced by interleukin (IL) 12-producing tumour cell vaccines but not IgG1 induced by IL-4 vaccine is associated with the eradication of experimental metastases. *Cancer Res* **58**: 5812–5817

Rowse GJ, Tempero RM, VanLith ML, Hollingsworth MA and Gendler SJ (1998) Tolerance and immunity to MUC1 in a human MUC1 transgenic murine model. *Cancer Res* **58**: 315–321

Ruiz PJ, Wolkowicz R, Waisman A, Hirschberg DL, Carmi P, Erez N, Garren H, Herkel J, Karpuj M, Steinman L, Rotter V and Cohen IR (1998) Idiotypic immunization induces immunity to mutated p53 and tumor rejection. *Nature Medicine* **4**: 710–712

Stewart LMD, Young S, Watson G, Mather SJ, Bates PA, Band HA, Wilkinson RW, Ross EL and Snary D (1999) Humanisation and characterisation of PR1A3, a monoclonal antibody specific for cell-bound carcinoembryonic antigen. *Cancer Immunol Immunother* **47**: 299–306

Taylor-Papadimitriou J, Peterson JA, Arklie J, Burchell J, Ceriani RL and Bodmer WF (1981) Monoclonal antibodies to epithelium-specific components of the human milk fat globulin membrane: production and reaction with cells in culture. *Int J Cancer* **28**: 17–21

Tempero RM, Rowse GJ, Gendler SJ and Hollingsworth HA (1999) Passively transferred anti-MUC1 antibodies cause neither autoimmune disorders nor immunity against transplanted tumours in MUC1 transgenic mice. *Int J Cancer* **80**: 595–599

Verhoeyen ME, Saunders JA, Price MR, Marugg JD, Briggs S, Broderick EL, Eida SJ, Mooren ATA and Bradley RA (1993) Construction of a reshaped HMFG1 antibody and comparison of its fine specificity with that of the parent mouse antibody. *Immunol* **78**: 364–370

von Mensdorff-Pouilly S, Gourevitch MM, Kenemans P, Verstraeten AA, Litvinov SV, van Kamp GJ, Meijer S, Vermorken J and Hilgers J (1996) Humoral immune response to polymorphic epithelial mucin (MUC-1) in patients with benign and malignant breast tumours. *Eur J Cancer* **32**: 1325–1331

von Mensdorff-Pouilly S, Verstraeten AA, van Kamp GJ, van Uffelen K, Snijdewint FGM, Paul MA, Meijer S, Kenemans P and Hilgers J (2000) Survival in early stage breast cancer is influenced by natural humoral immune response to polymorphic epithelial mucin (MUC1). *J Clin Oncol* **18**: 574–583

Zhang K, Sikut R and Hansson GC (1997) A MUC1 mucin secreted from a colon carcinoma cell line inhibits target cell lysis by natural killer cells. *Cell Immunol* **176**: 158–165

*© 2000 Cancer Research Campaign*
